# Halogenating Enzymes for Active Agent Synthesis: First Steps Are Done and Many Have to Follow

**DOI:** 10.3390/molecules24214008

**Published:** 2019-11-05

**Authors:** Alexander Veljko Fejzagić, Jan Gebauer, Nikolai Huwa, Thomas Classen

**Affiliations:** 1 Institute for Bio- and Geosciences I: Bioorganic Chemistry, Forschungszentrum Jülich GmbH, D-52426 Jülich, Germany; 2 Institute of Bioorganic Chemistry, Heinrich Heine University Düsseldorf, D-52426 Jülich, Germany

**Keywords:** bromination, chlorination, pharmaceuticals, active agent synthesis, biocatalysis, haloperoxidase, halogenase

## Abstract

Halogens can be very important for active agents as vital parts of their binding mode, on the one hand, but are on the other hand instrumental in the synthesis of most active agents. However, the primary halogenating compound is molecular chlorine which has two major drawbacks, high energy consumption and hazardous handling. Nature bypassed molecular halogens and evolved at least six halogenating enzymes: Three kind of haloperoxidases, flavin-dependent halogenases as well as α-ketoglutarate and *S-*adenosylmethionine (SAM)-dependent halogenases. This review shows what is known today on these enzymes in terms of biocatalytic usage. The reader may understand this review as a plea for the usage of halogenating enzymes for fine chemical syntheses, but there are many steps to take until halogenating enzymes are reliable, flexible, and sustainable catalysts for halogenation.

## 1. Still Up-to-Date—Halogens in Active Agents

For the discovery of new active agents, synthetic chemists frequently look into natural compounds and deduce lead structures and functionalities for the assembly of active agent libraries. Although most natural compounds are not halogenated, halogenation is spread over virtually all classes of secondary metabolites. Most of the halogenated natural compounds are of marine origin, while some are found in plants and insects as well [1]. Halogens appear in some form in 40% of all drugs being tested in clinical trials [2,3,4]. In addition to the fact that halogenations are an important structural motifs in natural substances and thus also in the resulting active substances, halogenations play a major role in the synthesis of many active substances. In the following, we want to figure out what is so special about the simple halogen moieties within molecules and reactions that make them so desirable, although the synthesis is very energy-demanding and carried out with toxic molecular halogens such as chlorine gas. In the second half of this review article we would like to show how nature realizes halogenations enzymatically and where we stand technologically to employ them as tools. In recent years, these enzymes have become even more prominent and the various scientific advances in this field have already been presented several times in an overview. These reviews also provide an up-to-date overview of the different enzymes, their substrate scope and biotechnological developments as well as the diversity of halo-compounds from all kingdoms of life [5,6,7,8,9,10,11,12,13,14]. The aim of this review is—among other things—to include a further point of view. In addition to the accurate arguments on the toxicity of elemental halogens and the cost-effectiveness of halide salts, a closer look at the actual costs of chlorine gas production was included, as well as a clear presentation that chemical halogenating reagents are all based on the provision of these halogen gases. In addition, the most recent achievements for industrial applications e.g., by up-scaling processes, but also the distribution of these enzymes, as well as the break with assumed dogmas, such as conserved structural motifs, were taken into account.

### 1.1. Electronical Properties of Halogen Moieties

**Figure 1:**
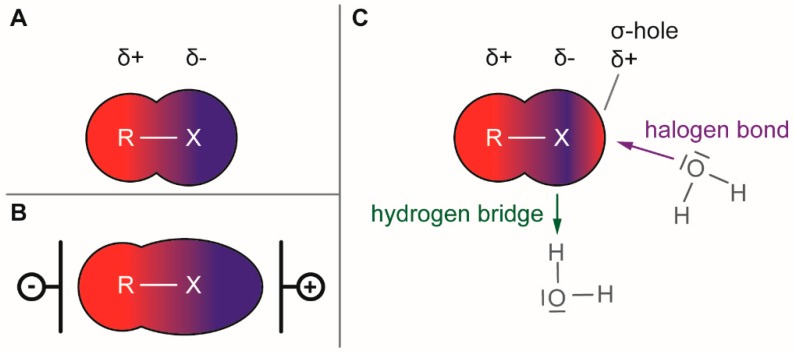
Schematic representation of electron distribution in halogens. (**A**): Latent polarization of a carbon-halogen bond. (**B**): Polarizability of large halogens (Br, I) bonded with a carbon. The external electrical field, for example, caused by an approaching electrophile/nucleophile leads to the distortion of the electron density. (**C**): Schematic view on the “σ-hole”. The electron density is drawn to the carbon-halogen bond, with the strength gradually increasing with the size of the halogen (I > Br > Cl >> F). This anisotropic distribution of electrons in the outer orbitals of the halogen creates an area of higher electron density around the belt of the halogen, allowing interaction with electrophiles or H-bonds. Orthogonal to the direction of the bond is an area of electron deficiency, creating a partially positively charged area in the halogen, allowing for nucleophilic attacks, commonly called “σ-hole”.

**Figure 2:**
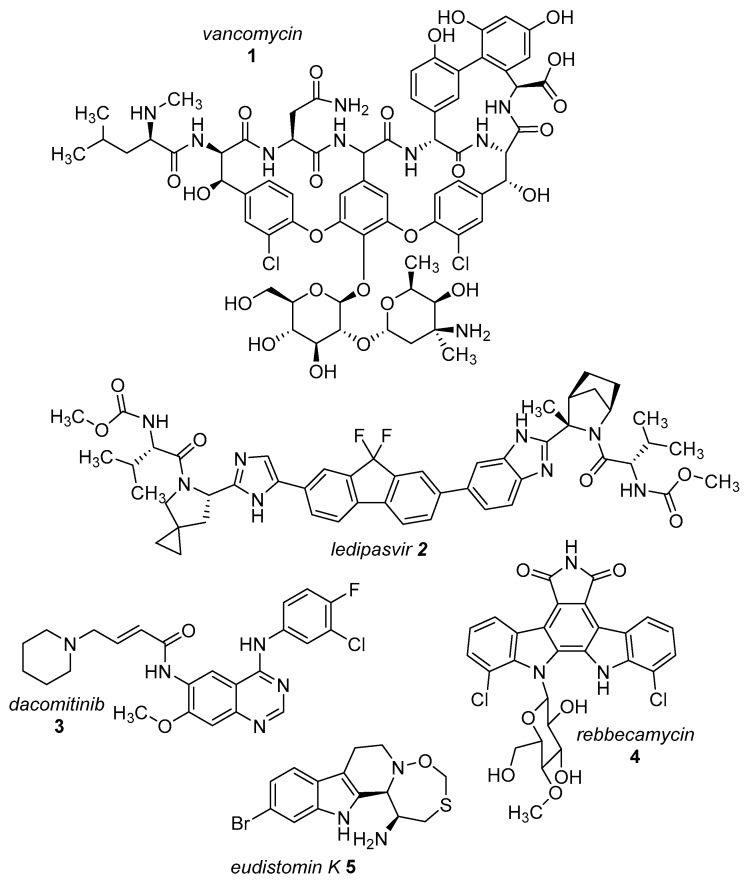
Examples for halogenated active agents.

The presence of a halogen (Cl, Br, I) usually increases the bulkiness of a compound, blocking for instance active site pockets or increases membrane permeability, relevant for oral absorption, and blood–brain barrier permeability. Besides their bulkiness, halogens exhibit extraordinary effects on the polarization of a compound. On the one hand, the halogens of the upper periods (F, Cl, Br) have a large electronegativity, which leads to a considerable latent polarization in the molecule (see Figure 1A). On the other hand, the polarizability increases with increasing period, so that interactions with soft nucleophiles or electrophiles in particular are promoted (see Figure 1B). Although the latent polarization is depicted in Figure 1A as a homogeneous gradient the model must be refined. Due to the p-orbital architecture there is a hole in the electron density opposing the binding partner of the halogen which is called the σ-hole (Figure 1C). Considering this σ-hole, it offers the option to interact with heteroatoms (O, S, N) by so-called halogen bonds as well as hydrogen bonds [15]. The ability to form halogen bonds has been the focus of several pharmacologically-oriented groups in the past years, as it can serve as an alternative non-covalent interaction between atoms (see Figure 1). For a detailed insight into the nature and characteristics of halogen bonds, as well as their possible impact on drug discovery in the future, see the corresponding articles [4,15,16,17,18,19]. The importance of halogens for biological activity of compounds can be profound. Vancomycin (**1**, Figure 2), an antibiotic, was shown to exhibit 30% to 50% less activity, based on the chlorine substituents missing, which is remarkable considering how small the portion of the halogens with respect to the entire vancomycin molecule is [20].

In terms of drug discovery, halogen substituents are regularly found in promising drug candidates with 35% in the discovery stage, while they appear in 36% of the candidates in clinical phase II and 26% in the drugs launched into the market (data from 2014) [16]. This trend shows that halogens play an important role in the field of drug design and discovery, and usually find their way to the final product assigned for treatment. In the following paragraphs, relevant halogens and some associated drug candidates containing halogen atoms will be discussed regarding their characteristic effects on bioactivity.

The most prominent halogen introduced into active agents is fluorine with 57% [3]. Due to its similar size compared to hydrogen and the extreme electronegativity, C–F bonds are polarized in a distinctive manner and render fluorine a weak halogen bond acceptor in contrast to be a good hydrogen-bond acceptor [21,22]. The covalent fluorine bond is very strong (456 kJ/mol for CF_4_), so that these bonds can only be cleaved under extreme and costly conditions in the body, if at all [23]. This increases the half time of active agents within the body (and environment) compared to their non-fluorinated pendants. Besides the electronic effects of fluorine within a molecule, fluorine also provides stereochemical properties which is summarized as fluorine *gauche* effect. Briefly, it can be described as a non-bonding weak interaction of the fluorine orbitals and other interacting partners. This reduces the degrees of freedom in rotation and this determines the conformation of a particular fluorinated molecule or guides reaction pathways. A review concerning this topic can be found in reference [24]. Apart from altering molecular characteristics, ^18^F is used as a common radioactive isotope label for in vivo study of protein function and enzyme catalysis [25]. Of all halogenated active agents, ledipasvir (**2**, see Figure 2) is one of the top-selling drugs, administered for the treatment of hepatitis C. Another important compound is dacomitinib (**3**), a single-fluorinated drug, which has been in clinical trials for the treatment of non-small-cell lung cancer [26].

Chlorine is the second prevalent halogen with 38% in halogenated drugs. Due to its increased size, it is a moderate halogen bond acceptor, while still being stable when being introduced into a carbon bond (327 kJ/mol for CCl_4_) [23]. Its presence in a compound alters volume and shape, allowing for positioning in deep cavities within proteins. These characteristics make it an interesting option for the functionalization of heterocycles. One of the most prominent chlorine-based natural compounds is rebeccamycin (**4**), a weak topoisomerase I inhibitor, which showed significant antitumor properties [27].

Brominated compounds are rarely found in drugs, making up only 4% of all halogenated compounds. This seems contradictory at first, as most halogenated compounds originate from marine organisms and are brominated despite chlorine being the more abundant halogen in water. Due to the lower polarization of the carbon-bromine bond and the extended bulkiness, bromine usually forms longer and thereby more labile bonds, not suitable for most drug candidates for a proper inhibition (272 kJ/mol for CBr_4_) [23]. These characteristics however allow an easier oxidation of bromine and consequently an easier incorporation into molecules, compared to chlorine. Although there is a prevalence of chlorinated and fluorinated active agents in pharmacology, some brominated compounds are known to display relevant bioactivity like eudistomin K (**5**), viable for the treatment of polio and herpes [28].

Iodine is the rarest halogen used (1%), commonly exploited for the synthesis of the active agents. Having a higher size and lower electronegativity, its bonds formed with carbon atoms are more labile than those of bromine, being easily cleaved off. Iodine is, therefore, preferably suitable for short-lived applications. An example of the use of iodine in medicine is radioactively labelled ^124^I in positron emission tomography (PET) as a tracer [29].

### 1.2. Halogens as Synthetic Tools

Both, bromine and iodine, are rare as functional moieties in active agents due to their labile covalent bonds. But it is precisely these properties that make halogens of higher periods valuable instruments for the synthesis of active substances.

A patent application for the production of hypohalous acids was applied for in 1944. *C. C. Crawford* and *T. W. Evans* described a process to obtain halide-free solutions of hypochlorous acid. This halogenating reagents were used in industrial applications to produce e.g., halohydrins from unsaturated organic compounds [30]. In 1993 another patent to produce concentrated slurries of sodium hypochlorite [35% (*w*/*v*)] was accepted [31]. They describe a process for highly pure hypochlorite slurry production. All the processes have the same starting materials in common. The first step is the solvation of molecular chlorine in water to get hypohalous acid (**6**) or the solution of sodium hydroxide and chlorine in water to end up with sodium hypochlorite. However, contaminations of sodium chloride and remaining sodium hydroxide occur in most processes that are carried out in industrial scale. The chlorine is hereby acquired by the chloralkali process where the electrolysis of sodium chloride produces molecular chlorine gas. Similar processes are state-of-the-art for the production of sodium bromate, which has the drawback of being a strong oxidizing agent [32,33], but can be used for the bromination of aromatic compounds [34]. The production of stable hypobromous acid is rather difficult because it easily oxidizes to bromate. Here, the production is carried out starting from hypochlorous acid or a modified chlorite [35].

**Figure 3:**
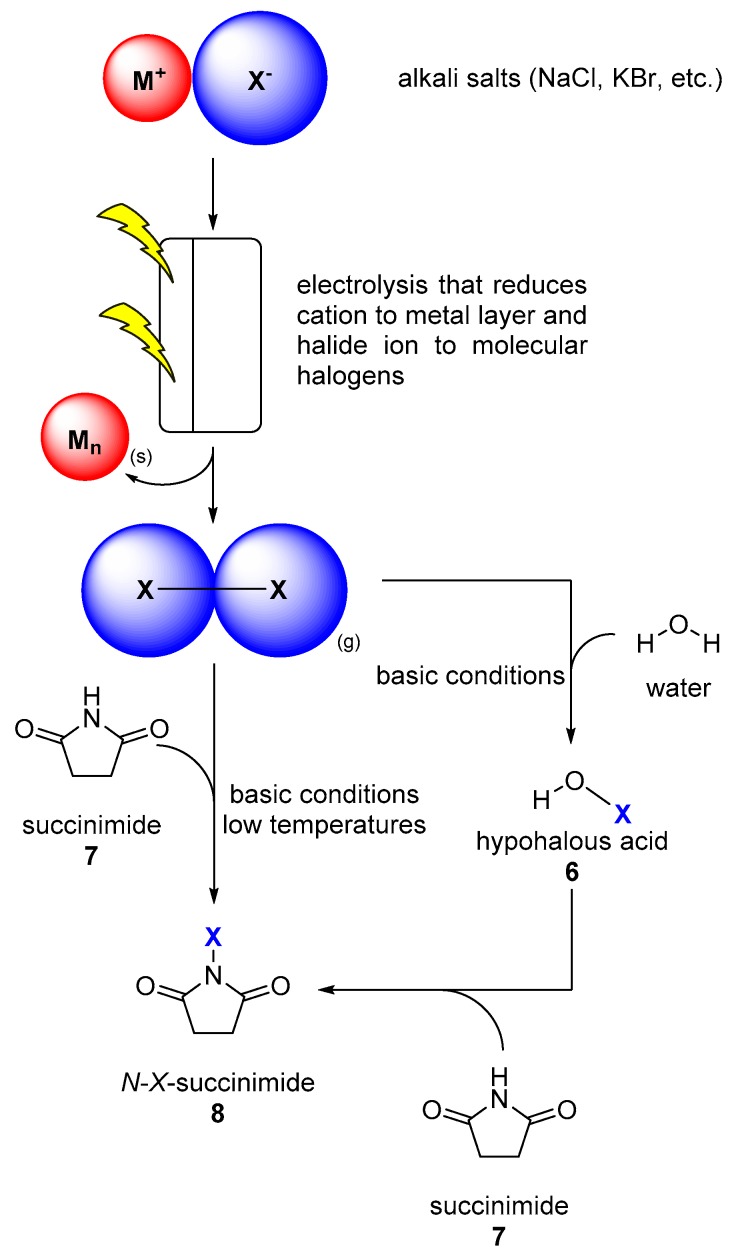
Workflow for the provision of halogenating reagents from alkali salts. The electrolysis process thus produces molecular halogens (X_2_), as well as hypohalous acids (HOX, **6**) and *N*-halogenated succinimides (NXS, **8**) in further steps.

More common halogenating agents are *N*-bromo-succinimide (NBS) and *N*-chloro-succinimide (NCS). Interestingly, even these reagents are synthesized from molecular halogens or hypohalous acids [36]. As a conclusion, it is now rather obvious, that all halogenating reagents have their origin in molecular halogen gases that are produced by cost-intensive procedures like halogen alkali electrolysis from halide salts (Figure 3).

**Figure 4:**
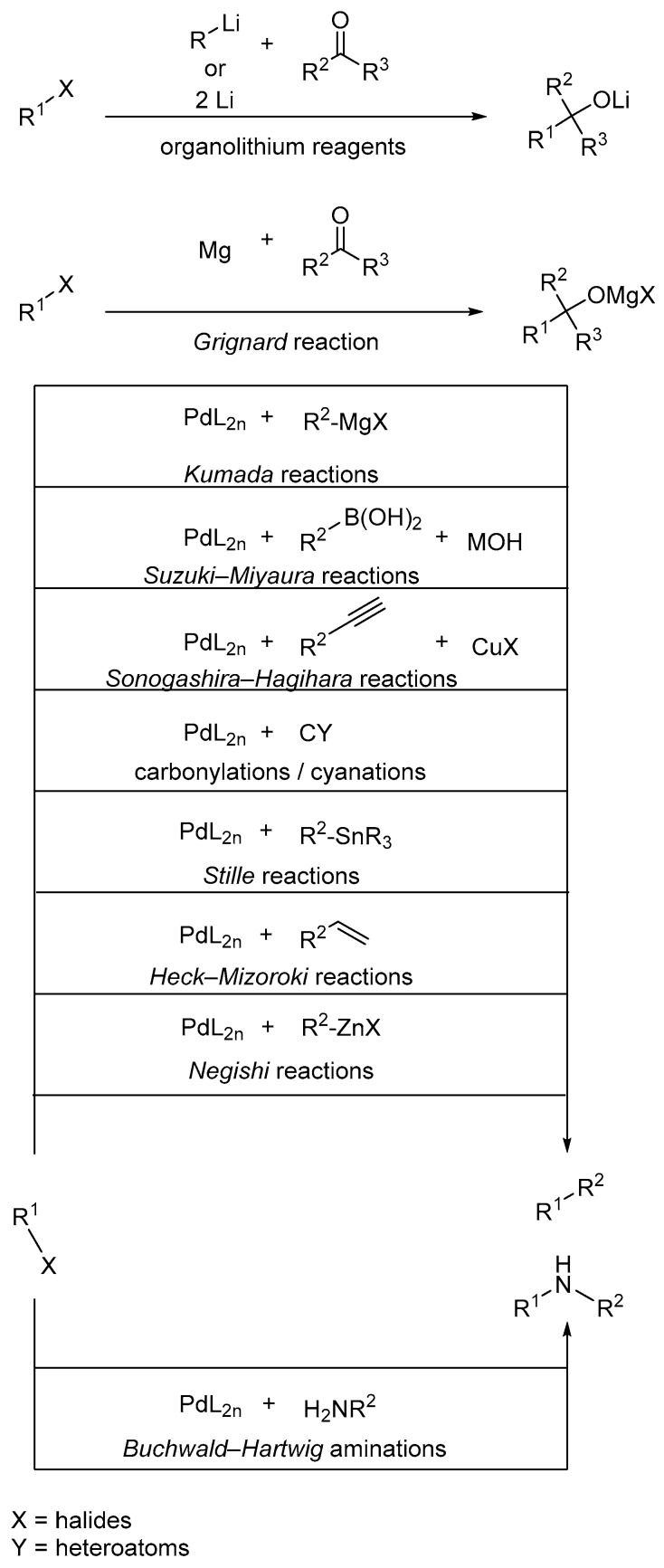
Most common reactions in organic synthesis exploiting halogen moieties. [37,45,46,47,48] Besides organolithium reactions as well as Grignard/Barbier reactions all of them are Pd-based, but can in many cases be substituted by other transition metals such as nickel.

**Figure 5:**
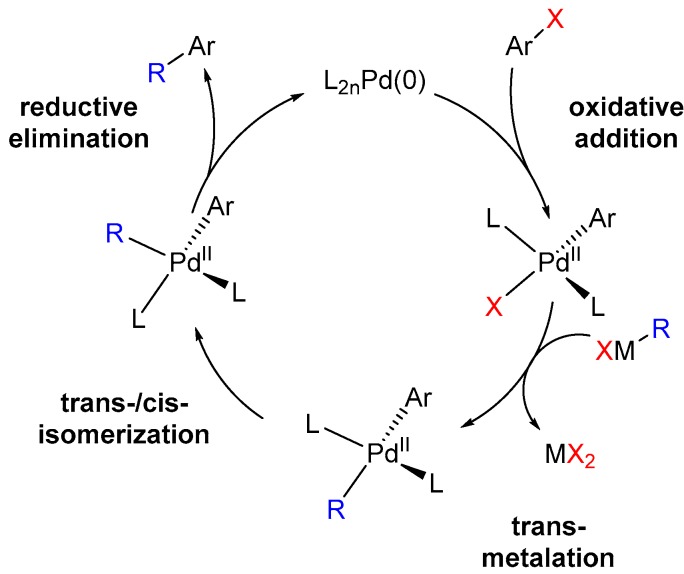
Scheme of the steps in cross-coupling reactions. After oxidative addition of the organo-halogen species, the transmetalation occurs. The ligands start rearrange before reductive elimination to the final product is carried out and the catalyst is regenerated.

Having these halogenated building blocks at hand, further synthetic steps can follow to build up active agents. Not only in academia but also in industry, the synthetic tool in terms of cross-coupling reactions is one of the most common C–C- and C–Y bond formations (Y is in this case N, O, S). With the use of different transition metals and activated carbon components, it is possible to generate large bioactive natural products and their derivatives. One prominent example is the use of palladium for the selective preparation of arenes and heterocyclic scaffolds with different substitution patterns [37]. However, also non-noble transition metals like copper [38], nickel [39], and nowadays even iron [40,41,42] are firmly anchored as suitable catalysts. Besides the high chemoselectivity, a profound functional group tolerance is a main advantage of these kind of reactions. Therefore, it is not surprising that industry has established approaches to produce pharmaceuticals and fine chemicals at the kilogram scale [43,44]. The following Figure 4 gives an overview of the most popular metal catalyzed named reactions, that slightly differ in their reactive moieties for both products or starting materials [37,45,46,47,48]. However, the catalytic cycle and thereby the reaction mechanism is very similar for all (Figure 5). Finally, conversions such as the *Appel* reaction and the *Hell-Volhard-Zelinsky* reaction, in which functional groups such as alcohols are converted to haloalkanes or carboxylic acids that become acid chlorides, must also be mentioned here.

### 1.3. Halogen Chemistry is Energy-Demanding

It is estimated that about 55% of chemical and 85% of pharmaceutical end products were processed with key components derived from the chloralkali electrolysis process [49,50]. These include hydrochloric acid to adjust the pH, or chlorinated solvents as part of the synthesis and subsequent isolation. However, this results in the production of the active compounds under hazardous conditions and high costs, due to toxic waste management. Using enzymes to halogenate pharmaceutical active compounds in a mild way and with a high efficiency is certainly a desirable aim for a greener chemistry. In general, the production and further processing of chlorine is mostly performed in the very same geographical region or facility in order to avoid the transportation of toxic and dangerous intermediates. This was reported for German companies and, presumably, this is also the case for other countries. The key component for halogenation (chlorine) is produced by electrolysis and is one of the most energy-consuming processes in the chemical industry. The process is responsible for about 2% of the total energy consumption yielding 5 million tons per year of chlorine in Germany [50,51]. Obviously, the energy reduction is an objective of the chloralkali industry, since 50% to 60% of the production costs is spend for the electrical energy [52].

## 2. Halogenating Enzymes

**Figure 6:**
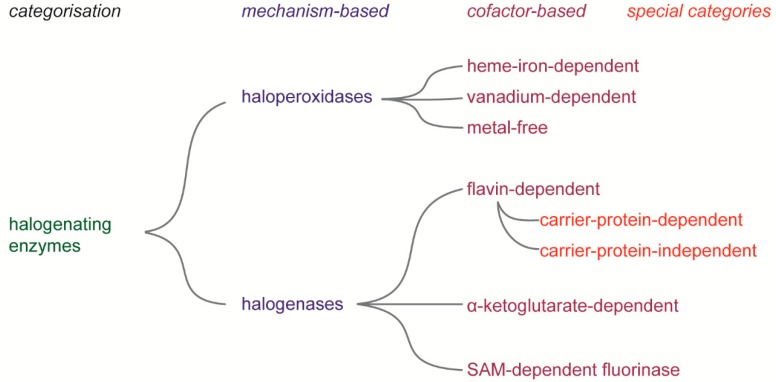
Overview on the categorization of halogenating enzymes.

Although halogenated natural compounds are rare and only found within the regime of secondary metabolism, at least six types of halogenating enzymes were evolved. Many were evolved from monooxygenases, since hypohalous acids are the core intermediate of catalysis in these halogenating enzymes. As diverse the origins of halogenating enzymes are as diverse is their classification. In Figure 6 we tried to give an overview on the categories of halogenating enzymes. Although often used synonymously, it can be differentiated between haloperoxidases and halogenases. The first group forms hypohalous acid from the respective halide and hydrogen peroxide via heme-iron-, vanadium-coenzymes, or even without any coenzyme. The hypohalous acid is set free for most of the enzymes and the very halogenation reaction takes place outside the active site. In contrast, the halogenases generate or simply use halonium species for the halogenation without the use of hydrogen peroxide.

### Haloperoxidases

Haloperoxidases were the first group of halogenating enzymes discovered in the past. Enzymes of this family catalyze the oxidation of a halide anion (X^−^) in presence of hydrogen peroxide to an oxidized halide form, usually believed to be the corresponding hypohalous acid. The class is further subdivided into three subclasses, the heme-iron-dependent, vanadium-dependent, and metal-free haloperoxidases or perhydrolases. In the following section, each class will be discussed briefly with biocatalytic examples, if they are known.

## 3. Heme-Iron-Dependent Haloperoxidases

The heme-iron-dependent haloperoxidases were the first and most intensively studied haloperoxidases. Back in the 1960s, an enzyme from the fungus *Caldariomyces fumago* (*Leptomyxes fumago*) was shown to be responsible for the halogenation of 1,3-cyclopentadion to the natural compound caldariomycin (**9**) [53]. Upon further investigation, it could be shown that it contained a heme-prosthetic group tethered to the enzyme by a distal cysteine ligand, very similar to the P450 monooxygenases [54].


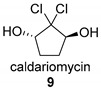


**Figure 7:**
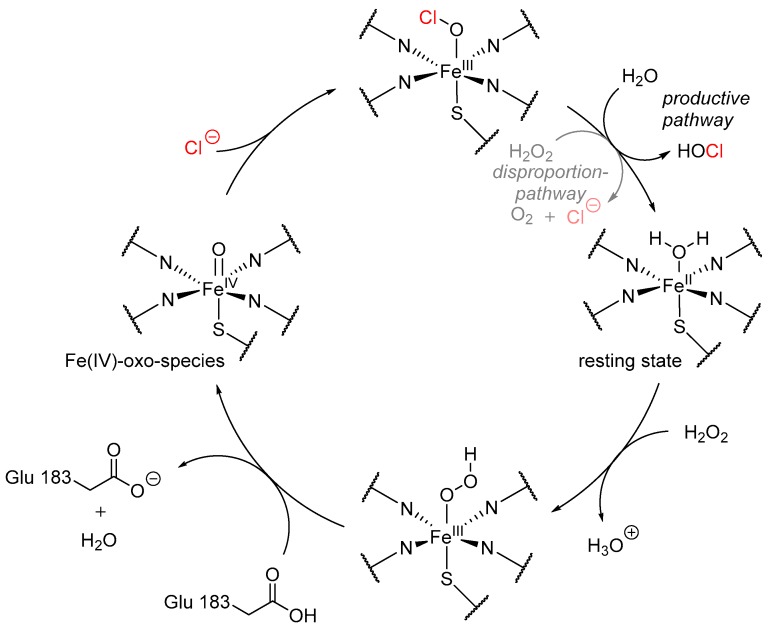
Proposed catalytic cycle of heme-iron-dependent haloperoxidases, shown on the example of CPO from *C. fumago*. In the resting state (3 o’ clock), water is bound to the heme-iron, which is subsequently replaced by hydrogen peroxide. After protonation of this complex by a catalytic glutamate (Glu183), water is eliminated, creating the actual active species, the Fe(IV)-oxo complex. A halide, in this case chloride, binds to the Fe(IV)-oxo species and is released as hypochloric acid, regenerating the heme-site by hydrolysis with water. Alternatively, another molecule hydrogen peroxide may attack, leading to the disproportion of the complex to molecular oxygen, water, and chloride [54,55].

The catalytic cycle (Figure 7) displays a key intermediate, the Fe^IV^-oxo-species, to oxidize chlorine to hypochlorite, which is released and may be attacked by an electron-rich substrate serving as an electrophile. In presence of excess hydrogen peroxide, this complex can alternatively decompose to molecular oxygen and chloride.

As the enzyme resembled characteristics from peroxidases as well as monooxygenases, it was classified as a heme-iron-dependent haloperoxidase and due to its ability to oxidize all halides besides fluorine was named chloroperoxidase. Recently it was revealed that actually two *Cf*-*cpo* genes within the *C. fumago* genome exist, sharing a high sequence identity and both being present in the secreted supernatant of its host [56]. Since its discovery, the enzyme was target of many mechanistic and biocatalytic studies. To much surprise, the formed hypohalous acid does not leave the active site freely, but is held back by amino acids placed in the halide entrance tunnel of *Cf*-CPO, allowing for regio- and enantioselectivity to a certain degree, mainly depending on the nature of the substrate [57]. Its major drawback, however, was the oxidative inactivation every heme-iron-containing protein suffers after exposure to oxygen as well as a high sensitivity for high hydrogen peroxide concentrations. As the genetic modification of the fungus can prove tedious, the application of this enzyme in biocatalysis might seem limited, however due to the fruitful work of Pickard et al., protocols are available for a reasonable production and secretion of the enzyme in the native host, *C. fumago* [58].

**Figure 8:**
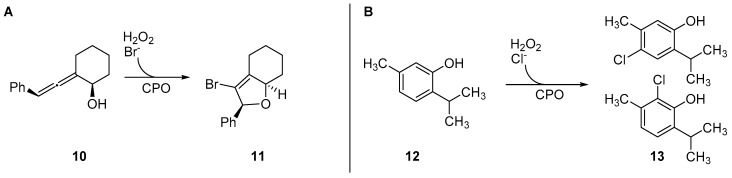
Example reactions of *Cf-*CPO involved in biocatalytic conversions of organic molecules (**A**): Cyclization of allenes (**10**) to the product **11** induced by halogenation with Br^−^ by *Cf-*CPO. (**B**): Unselective chlorination of thymol (**12**) by *Cf-*CPO.

As a catalyst, *Cf*-CPO was shown to be rather robust und allow a variety of different organic transformations, where some are not always bound to a halogenating step. It could be applied in cascade reactions with oxidases leading to halocyclization reactions of allenes (**10**) and even be immobilized for (semi-)continuous-flow bioreactors [59,60,61] (see Figure 8). It was used for the halogenation of phenolic monoterpenes like thymol (**12**) and carvacrol, excelling with drastically lower catalyst loadings (by five orders of magnitude) compared to chemical alternatives like Cu^II^-catalysis [59]. Furthermore, it was shown to be capable of halogenating *trans*-cinnamic acid and other unsaturated carboxylic acids, as well as catalyze enantioselective epoxidation of alkenes [62,63]. One bottleneck observed was the low substrate loading, impairing possible preparative applicability.

Besides chloroperoxidase from *C. fumago*, not many members of this subclass have been dealt with. The bromoperoxidases from *Pseudomonas aureofaciens* and *Penicillus capitatus* are other examples of such heme-iron-dependent enzymes [64,65]. However, beside classic characterization experiments, revealing similar properties to *Cf*-CPO such as high thermal stability and sensitivity to high hydrogen peroxide concentrations, no complex biotransformations were investigated with these enzymes, yet (see Table 1) [66].

## 4. Vanadium-Dependent Haloperoxidases

For several years after the discovery of heme-iron-dependent haloperoxidases, it was assumed that they are the only enzymes able to oxidize halides for the subsequent halogenation reaction. However, a new halogenating enzyme class was discovered in 1993 by van Schijndel et al. from *Curvularia inaequalis* using *ortho*-vanadate cofactor for the oxidation of halides [70,71]. Just two years later, a vanadate-dependent homolog from *Corallina officinalis* was crystallized [72]. These vanadium-dependent haloperoxidases became a popular research target as they were shown to exhibit high turnover numbers without suffering an oxidative inactivation and displaying a higher tolerance against hydrogen peroxide [73], In contrast to the heme-iron-dependent ones, however, they usually do not retain the formed hypohalous acid within the active site, leading to a freely diffusible strong oxidant. Resulting from this mechanistic aspect, random halogenations occur, even in the protein itself, leading to its destabilization and inactivation. Because of this free hypohalous species, the selectivity of the subsequent halogenation reaction is independent of the enzyme but from the electronic properties of the substrate. Most of the vanadium-dependent haloperoxidases originate from marine fungi and marine macroalgae (seaweeds) [74].

**Figure 9:**
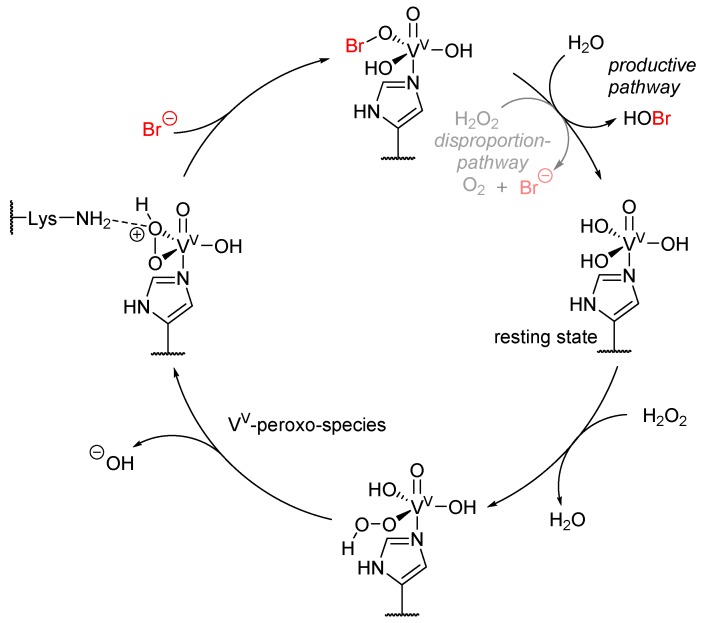
Proposed catalytic cycle of vanadium-dependent haloperoxidases. In its resting state (3 ‘o clock), vanadium contains four oxygen ligands, while the free coordination site is occupied by a catalytic histidine residue, resulting in a dative bond. In presence of hydrogen peroxide, a hydroxyl group is substituted by peroxide. Upon elimination of a hydroxide ion, a cycloperoxo-species is generated, which is stabilized by a catalytic lysine residue. This cyclic intermediate is opened by addition of a halide, in this case bromide, which can then be hydrolyzed by water, leading to the release of hypobromic acid, or in presence of another hydrogen peroxide molecule, be disproportioned to molecular oxygen and bromide. During catalysis, the vanadium does not alter its oxidation state (V) [55].

It is proposed that the catalytic cycle (Figure 9) forms a V^V^-peroxo-species as the key intermediate, where the halide is added and subsequently hydrolyzed to hypohalous acid. Identically to heme-iron-dependent haloperoxidases, the presence of hydrogen peroxide may lead to the disproportion to singlet oxygen and the halide [55].

One of the best-investigated representatives of this class is the vanadium-dependent chloroperoxidase from the phytopathogenic fungus *Curvularia inaequalis* [70,71,75,76,77]. Even in absence of the vanadium-cofactor, the enzyme is stable in its *apo*-form and can easily be transformed to the holo-form by external addition of *ortho*-vanadate [70]. Although the gene can be heterologously expressed in *E. coli* and activated with vanadate, it was reported that the amount of enzyme obtained was very low. As an alternative, *Saccharomyces cerevisiae* was used as a host, yielding 100 mg/l *apo*-enzyme [75]. Kinetic experiments lead to a k_cat_/K_M_ of 2.6 × 10^6^ m^−1^ s^−1^ for hydrogen peroxide and 5.1 × 10^7^ m^−1^ s^−1^ for bromide at pH 4.2, the optimal pH for bromoperoxidase activity [75].

**Figure 10:**
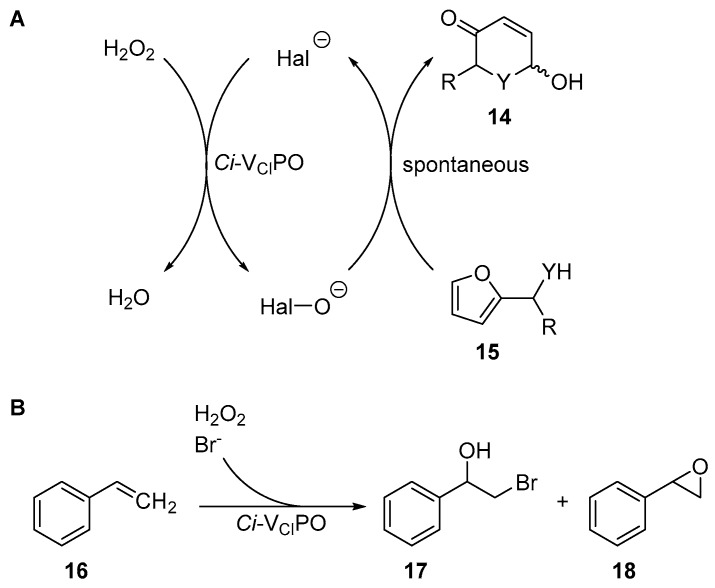
(**A**): *(Aza-)Achmatowicz* reaction transforming the furan **15** to the Michael-system [78] **14**. (**B**): Halofunctionalization of styrene (**16**) [77,79].

It showed stability at high temperatures (T_M_ of 90 °C) and tolerance against organic solvents like methanol, ethanol, and propan-2-ol (up to 40% *v*/*v*) [71]. *Ci*-V_Cl_PO was used as a hypohalogenite catalyst for the halogenation of phenols like thymol, while showing excellent stability towards hydrogen peroxide and organic solvents like methanol and ethyl acetate [76]. Furthermore, it was used for the mediation of *(Aza-)Achmatowicz* reactions in combination with cascades [78] and halofunctionalization reactions of aromatic and aliphatic alkenes like styrene and hexanol [77,79] (see Figure 10).

In contrast to the usually scarce selection of vanadium-dependent chloroperoxidases, many representatives of bromoperoxidases were researched in the past. One of the most prominent members of this group is the V_Br_PO from *Corralina officinalis*, a marine red algae. Similarly to the homolog from *C. inaequalis*, it excels with a high stability towards high temperatures up to 90 °C and in presence of organic solvents like ethanol, propanol, and acetone (up to 40% *v*/*v*) [72]. However, recombinant expression of the gene in *E. coli* BL21(DE3) proved difficult, as the amount of protein formed is high, but insoluble. Coupe et al. notably showed that by using a refolding procedure, 40 mg/L of active enzyme can be retrieved after expression and isolation [80].

**Figure 11:**
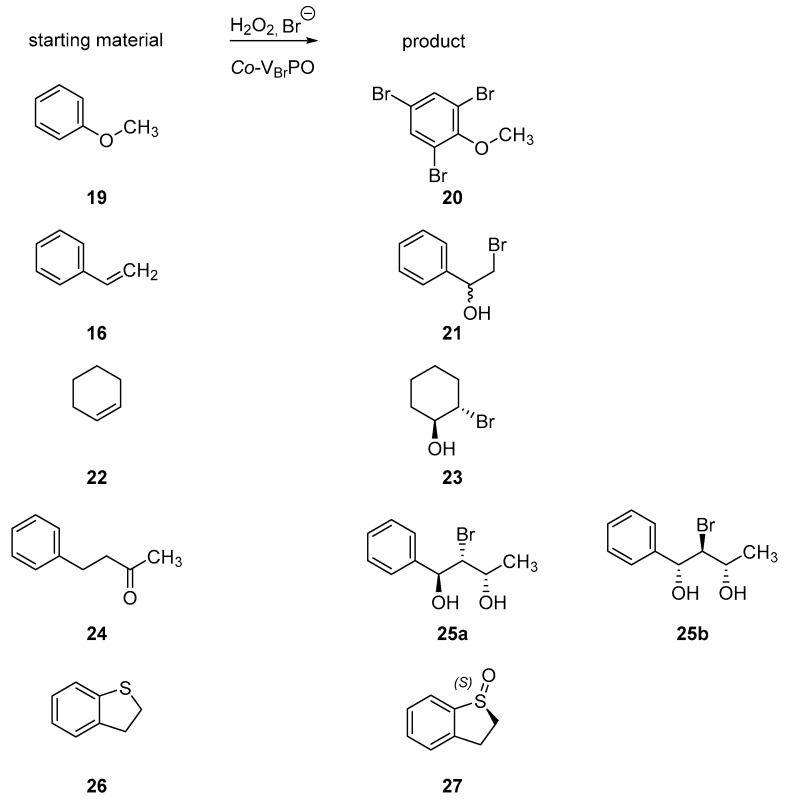
Selected reactions performed by the *Co*-V_Br_PO to illustrate the reaction spectrum [81].

The haloperoxidase was shown to accept a variety of substrates, like nitrogen-containing heterocycles, cyclic β-diketones, phenol, *o*-hydroxybenzyl alcohols, anisole (**19**), 1-methoxynaphthalene and thiophene in addition to alkene halogenations with styrene (**16**), cyclohexene (**22**) among others to yield various bromohydrins [81] (see Figure 11 and Table 2).

In most of the cases, no diastereoselectivity for the bromohydrin formation could be observed, except for the formation of bromohydrin from (*E*)-4-phenyl-buten-2-ol (**24**) [69]. Besides bromination reactions, haloperoxidases like the *Co*-V_Br_PO are able to catalyze sulfoxidations with 2,3-dihydrobenzothiopene (**26**), as well [82].

## 5. Metal-Free Haloperoxidases/Perhydrolases

Although oxidative halogenation reactions are dominated by (transition) metal catalysis in nature, a group of enzymes was identified catalyzing halogenation without any metal cofactor. These metal-free haloperoxidases or perhydrolases were found to require hydrogen peroxide and halides as well, while forming percarboxylic acids from carboxylic acids using a catalytic triad of serine, histidine, and aspartate [84,85]. Their striking resemblance to lipases has initiated a general debate over the nature of these enzymes, as their characteristics resemble hydrolases with a halogenating sub-activity. This has led to controversies whether the metal-free haloperoxidases are not simply lipase-like enzymes moonshining as haloperoxidases. In fact, several lipases were tested positively for haloperoxidase activity despite low turnover numbers [81].

**Figure 12:**
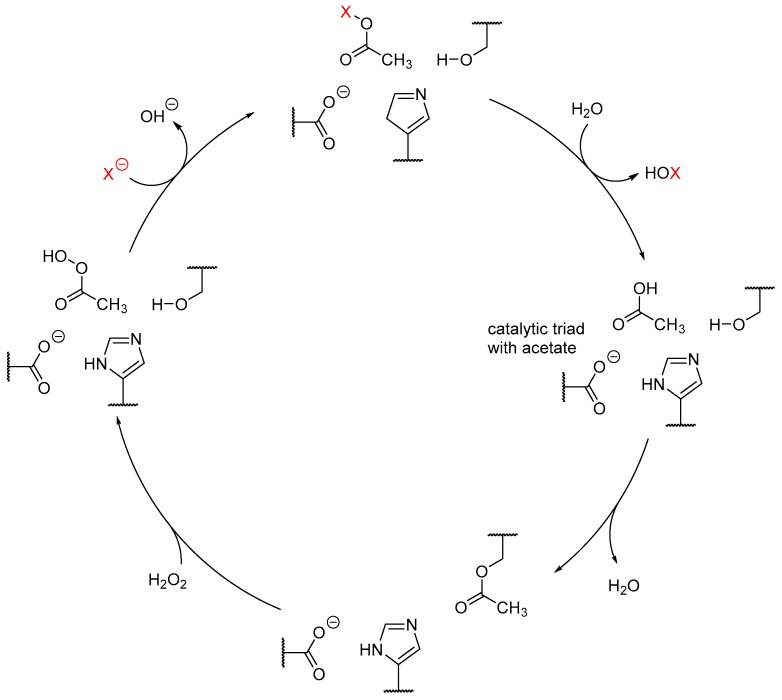
Proposed catalytic cycle of metal-free haloperoxidases/perhydrolases. This mechanism was compiled from several sources [81,85]. The catalytic cycle is adopted from the common hydrolase catalysis encountered in lipases and esterases, for instance. In presence of a carboxylic acid, in this case acetic acid, an ester is formed with the catalytic serin residue upon elimination of water (3 ‘o clock). In presence of hydrogen peroxide, the ester is cleaved, forming a percarboxylic acid. In the following step, a halide binds to the peroxoacid, which is hydrolyzed to the hypohalous acid, while the characteristic Ser-His-Asp triad is already regenerated.

The key-step in catalysis is the formation of a peroxo-acid from a carboxylic acid by hydrogen peroxide, which subsequently forms an acylhypohalide acting as the halogenating agent (Figure 12) [86].

**Figure 13:**
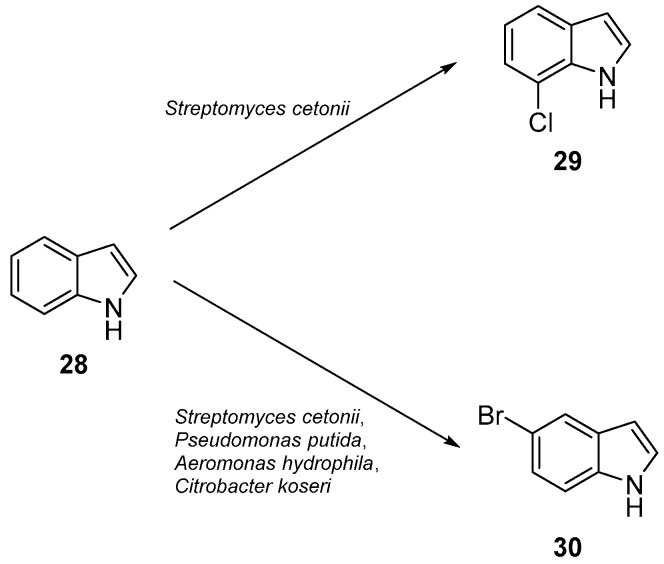
Halogenation of indole (**28**) nucleobase analogs according to Lewkowicz and co-workers [87].

Many examples for metal-free HPOs in biotransformations are not known. The majority of investigations of this enzyme class were focused on determining and expanding the tolerance of these enzymes to organic solvents and temperatures. One recent example of a bioorganic application was the halogenation of nucleobases and analogues [87] (see Figure 13).

### 5.1. Flavin-Dependent Halogenases

In addition to the long-known haloperoxidases, another class of enzymes has aroused much interest. It is suspected that flavin-dependent halogenases (FHals, Fl-Hals, or FDHs) evolved from monooxygenases that require flavin cofactors as well and, therefore, belong to the superfamily of flavin-dependent monooxygenases [88,89].

**Figure 14:**
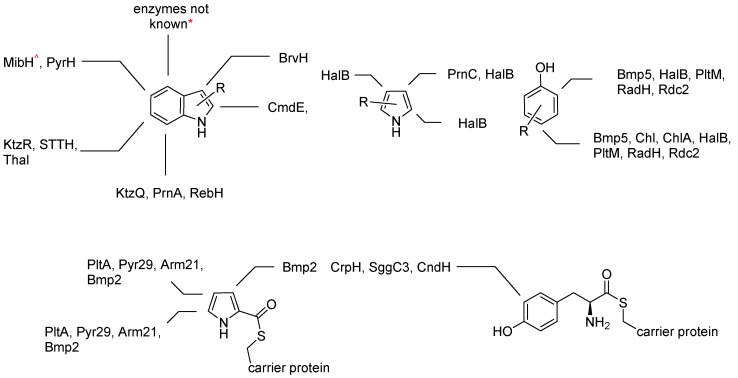
Regioselectivity of flavin-dependent halogenases and their dependency on carrier proteins. * Natural products with halogenations are known, but so far, no enzyme is characterized. ^^^ This tryptophan halogenase is one of the few examples that is carrier protein-dependent [99].

According to what is known so far, there are three natural target structures that can be addressed by FHals. The most studied and best understood group are the flavin-dependent tryptophan halogenases. In nature, there is the possibility to halogenate every position of the indole ring. Similar to this structure there is the group of flavin-dependent pyrrole halogenases and finally the flavin-dependent phenol halogenases (see Figure 14) [8]. The fact, that each and every position can be addressed by an individual enzyme demonstrates that FHals are selective halogenating catalysts in contrast to the majority of haloperoxidases. FHals must be differentiated according to the accessibility of their substrates. While a large number of these halogenases are involved in biosynthesis clusters of polyketides (PKS) and non-ribosomal protein synthesis (NRPS), some, such as tryptophan halogenases, can convert freely diffusible substrates and are not dependent on carrier proteins that activate or merely tether the substrate (Figure 14) [13].

For the application of this enzyme group, it is important to keep in mind that they need at least a two-component electron transport chain and therefore require a suitable flavin reductase [90,91,92]. In addition to the reductases that naturally belong to the biosynthesis clusters e.g., PrnF [93], applications with other reductases such as SsuE [91,94,95] or Fre [96,97] from *E. coli* have also been reported. To avoid the necessity of a second enzyme—the flavin reductase—or even a third enzyme for cofactor recycling, photochemical approaches are in the focus of current research in this field as well [98].

Figure 14 shows some representatives for the halogenation of the different positions of the different substrates (indoles [92,95,100,101,102], pyrroles [103] and phenols [104,105,106,107]), each with reference to the halogenating enzyme, the dependence on carrier proteins and the corresponding publication [108]. The halogenation of position four of indoles, as for example in 4-chloroindole-3-acetic acid, is known to date only from plants (*Pisum sativum*, *Lens culinaris*, *Vicia* sp., and in particular *Vicia faba*), as a growth hormone but no enzyme has yet been characterized responsible for its formation [7]. The publications e.g., by Shepherd et al., the review of Latham et al. and other publications also show various mutants that led to changes in regioselectivity and substrate scope [8,96,100,109,110].

**Figure 15:**
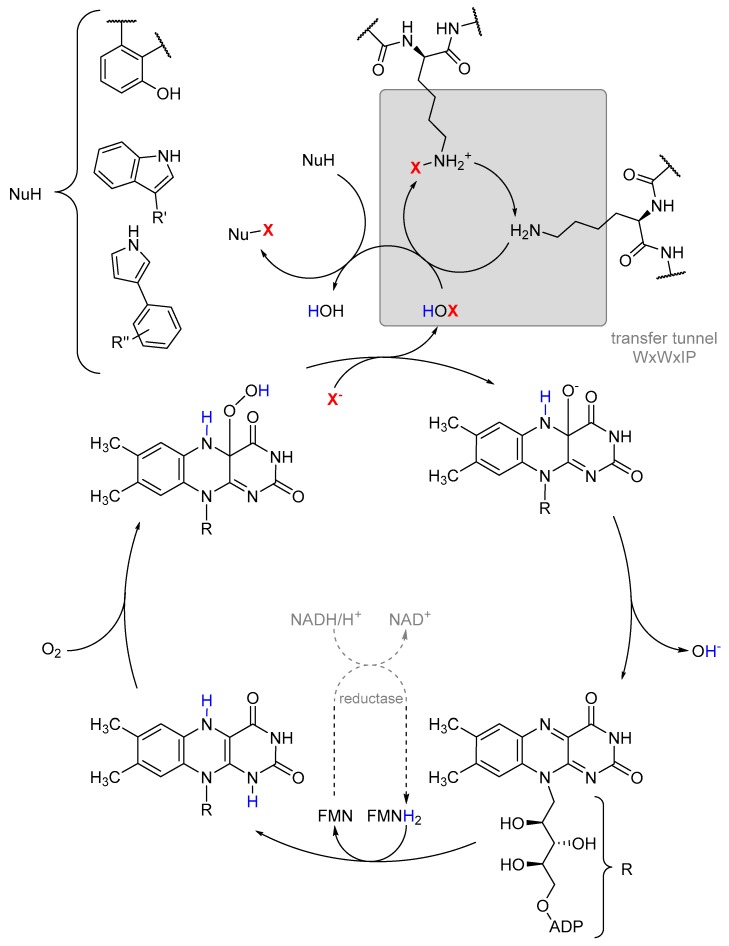
Catalytic cycle of halogenation by flavin-dependent halogenases [96,115].

A lot of these enzymes that are dependent on carrier proteins produce well-known secondary metabolites like rebeccamycin (**4**) and vancomycin (**1**) but also a plethora of less investigated biosynthetic pathways [133]. The most important difference in the mechanism between flavin-dependent monooxygenases and halogenases is the conserved motif of two tryptophanes, one isoleucine and proline. This 10 Å long tunnel [89], first found in PrnA, serves to spatially separate the activated peroxy-flavin FAD(C4α)–OOH from the substrate binding site and thus prevents oxygenation [114,116,128]. After generating the hypohalides, a conserved lysine transfers the electrophilic chlorine as chloramine from the former peroxy-flavin to the substrate (Figure 15) [134].

For the phenol halogenase the mechanism is proposed to be slightly altered. The phenolic hydroxyl group is deprotonated by an aspartate within the active site increasing the nucleophilicity of the enol α-carbon [130]. Based on these conserved motifs and the assumed reaction mechanism some putative halogenases have already been found and annotated. Recently even a viral halogenase VirX1 from cyanophages was discovered, which is the first FHal capable of in vitro iodination and stands out due to its broad substrate spectrum and preferred iodination [135].

Although the community has so far agreed that the preserved motif of separation tunnel and anchor lysine seems to be essential for the activity, current research shows a further class of flavin-dependent halogenases which lack these structural elements completely. One of these examples is the halogenase KerK that is under investigation by Piel and coworkers but has not yet been published except as a poster presentation on Biotrans 2019 in Groningen, the Netherlands [136].

In addition to the advantages of high regioselectivity and thus only few by-products, there are also some disadvantages in the use of this enzyme group. The low conversion rates speak against large-scale application and expression problems often occur. Many of the proteins produced in *E. coli* BL21(DE3) end up in the insoluble fraction as inclusion bodies. To deal with this issue, strains with co-expression of chaperones are used regularly (Table 3). The overall stability of these proteins also needs further optimization to be applicable in biocatalysis. As a promising result Kemker et al. the tryptophan halogenases were successfully scaled up in terms of a biocatalytic process employing immobilizing the enzymes by cross-linked enzyme aggregates (CLEAs). This yielded l-7-bromotryptophan on the gram scale [137].

### 5.2. α-Ketoglutarate-Dependent Halogenases

**Figure 16:**
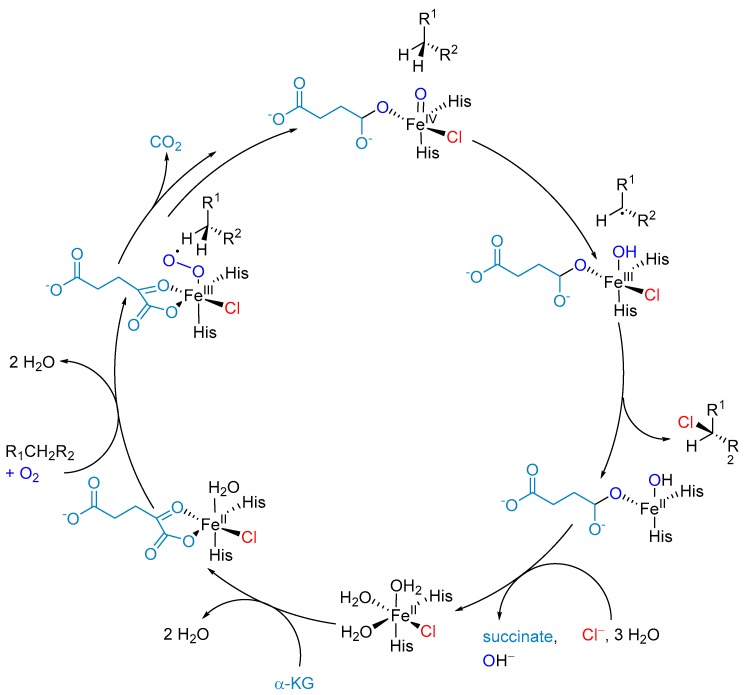
Proposed mechanism for halogenation reaction by Fe(II)/αKG-dependent halogenase via a radical C-H functionalization [142]. The highly reactive Fe(IV)-oxo (haloferryl) intermediate is produced by decarboxylation of αKG to succinate through an oxygen attack. Subsequently abstraction of a hydrogen-atom from the substrate leads to an energetically favourable rearrangement towards Fe(III). Rebound reaction with chloride was shown to depend on the distance and orientation of the substrate [143]. The catalytically cycle is re-established by the hexa-coordinated Fe(II) with water molecules, chloride and histidine.

Table 4 shows different natural products that are formed by the iron(II)-α-ketoglutarate-dependent (Fe/αKG)-halogenases. Despite the huge variety in the product structures they share one common feature, which is the halogen at a sp^3^-carbon centre. Hence, the Fe/αKG-halogenase is not limited to nucleophilic substrates like the previous described enzymes. They belong to the Fe/αKG-dependent oxygenase superfamily. The superfamily is known for different transformations such as hydroxylation [138], halogenation [139], desaturation [140], or can be used for the production of ethylene [141]. They all share a structurally conserved metal-binding motif, which in the case of the halogenase developed an active centre that is eventually able to bind a haloge n [139]. The proposed catalytic mechanism of Fe(II)/α-KG-dependent-halogenase is illustrated in Figure 16.

Based on the proposed radical C-H functionalization two classes of enzymes have so far been identified. The first such reported Fe/αKG-dependent halogenase is the tailoring domain SyrB2 of the multimodular nonribosomal peptidsynthetase (NRPS) from *Pseudomonas syringae* pv. *syringae* B301D [133,144]. These NRPS-associated halogenases produce a diversity of secondary metabolites such as the chlorinated biosurfactant syringomycin E (**31**), which is characterized by a selective monochlorinated threonine in its structure [128,139]. Another example is the highly selective di- and trichlorination of solely one of the diastereotopic methyl groups of leucine by a combination of BarB1 and BarB2, which serves as a precursor for the natural compound barbamid (**32**) in the marine cyanobacteria *Lyngbya majuscula* [142,153].


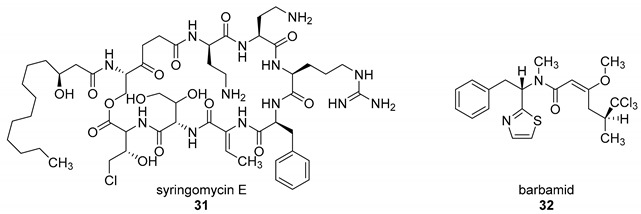


**Figure 17:**
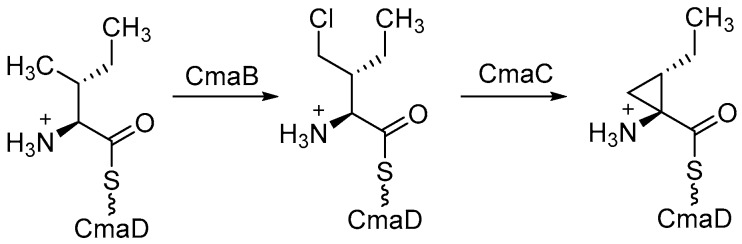
Schematically example for formation of cyclopropane initiated by CmaB through halogenation.

Recently Moosmann et al. identified different αKG-halogenase homologues and their natural products that are produced via a NRPS (non-ribosomal peptide synthetase) pathway. The halogenase were identified by screening the genomic sequence of the cyanobacterium *Fischerella* sp. PCC 9339 based on feature comparison. Using this approach, the authors were able to distinguish between a Fe/αKG oxygenase and a corresponding halogenase [143,154]. However, large NRPSs characteristically bind their substrates through an aminoacetylated peptidyl-carrier protein and have a narrow substrate scope [144,155]. Furthermore, they generally showed a low total turnover number, which may result from the well-known autoxidation of Fe(II) to Fe(III) and hence an auto-inactivation of the enzyme [145,156]. In case of SyrB2, total turnovers of 7 ± 2 were observed [133,144] This limits the possibility to modify the enzymes in order to use them as suitable biocatalysts for different unnatural substrates. With the discovery of a new Fe(II)/αKG-dependent halogenase (WelO5) by Hillwig and Liu, it was possible to expand the class towards unbound substrates. WelO5 is capable of late stage halogenation in a regio- and stereoselective manner of different derived isoprenoid-indole alkaloids in the cyanobacterium *Hapalosiphon welwitschii* (see Table 4) [137,148]. WelO5 showed also a higher robustness and catalysis of approximately 75 turnovers in total [137,148]. Strategies such as adding the cosubstrates consecutively or adding antioxidants like catalases or DTT could increase the turnover number. The narrow substrate scope of WelO5 was tailored in order to have an increased substrate scope like the homolog AmbO5 [138,149]. Most modifications were at the external helix, which is responsible for closing the entry of the active site upon binding of the substrate. It can be assumed that the helix is partially involved in the substrate recognition and specificity [138,149]. A recent publication from Hayashi et al. showed a WelO5 variant with a reshaped active site that led to improved kinetics and an expanded substrate scope, which applies beyond the native indole alkaloid-type substrates [141,152]. This provides the possibility for targeted enzyme-engineering and a basis for further improvements in substrate scope. One possibility is the establishment of nitration and azidation as already shown for SyrB2 [157]. In this regard, it has been shown that WelO5 is able to incorporate the unnatural halide Br^−^ [158]. One drawback of engineering Fe/αKG-dependent halogenases is the hydroxylation as a competitive side reaction [155]. Mitchell et al. used this approach backwards and modified a monooxygenase SadA towards a halogenase [159]. This serves as a proof of concept that with increasing understanding of the reaction mechanism and the involved amino acids the superfamily of monooxygenase can be used as a versatile toolbox in biotechnology. In the future, this may lead to the use of different variants of the very same enzyme for different transformations. Table 4 shows an overview of different characterized Fe/αKG halogenases and their main published features. Excluded are, for example, halogenase modules of NRPS, where the halogenation is necessary for the subsequently formation of cyclopropane such as in case of CurA [160] or CmaB (see Figure 17) [161].

### 5.3. Fluorinase

**Figure 18:**
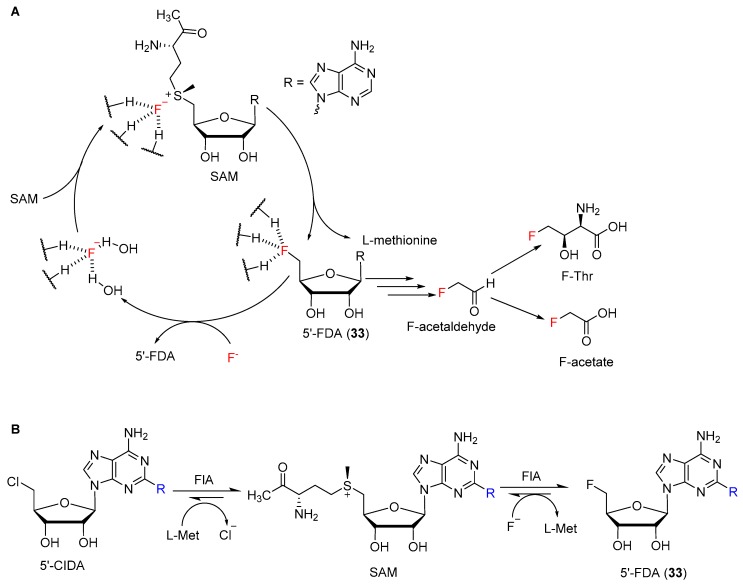
(**A**) Schematically sequential mechanism for the F–C bond formation catalysed by the fluorinase from *S. cattleya* and some products of subsequently cascade reaction. Dashed lines represents hydrogen bonding contacts with amino acids in the active pocket or water. (**B**) Reaction scheme of fluorinase-mediated trans-halogenation. Rest R marks position of usual derivation.

In contrast to the other described enzymes, the diversity of natural products in case of the fluorinase stem from only one characterized enzyme class to date. The involved enzymes are *S*-adenosylmethionine (SAM) dependent. The first characterized representative was FlA (5′-fluoro-5′-deoxyadenosine synthase) from ** [162]. The overall family of this enzymes is also able to chlorinate or hydroxylate SAM, as described in detail elsewhere [163]. Within the catalytic cycle fluoride acts as a nucleophile in a S_N_2-reaction, where it attacks the 5′-carbon of SAM-ribose [164]. In order to act as a nucleophile, fluoride requires to lose its solvation shell. This is achieved in a two-step desolvation with a combination of electrostatic stabilization and hydrogen bonding. In the first step, fluoride is binding to the active site and exchanges water molecules of its shell in order to form hydrogen bonds with the enzyme. Upon binding of SAM the desolvation of fluoride is complete. The electropositive 5′-carbon attached to the sulfur group in SAM coordinates with the fluoride [165,166]. This electrostatic stabilization facilitates the nucleophilic attack of the fluoride and C−F-bond formation of the reactive 5′-fluoro-5′deoxyadenosine (**33**, 5′-FDA) intermediate [166]. Subsequently, 5′-FDA (**33**) is further metabolized in order to generate a variety of compounds as shown in Figure 18A [162]. However, this also represents a major obstacle for the application of these enzymes to unnatural small organic molecules, since the product formation follows a cascade of enzymatic steps. Eustáquio et al. tried to use this enzyme for the production of fluorosalinosporamide, an unnatural analog of salinosporamide, which is fluorinated rather than chlorinated, however, the yield was moderate [167]. Nevertheless, different approaches have been implemented to increase the substrate scope and use the enzyme as rather flexible tool for medical applications. Besides the ability to fluoride compounds, fluorinase is also able to exchange a chloride at the 5′-carbon of the SAM ribose ring by a fluoride and form 5′-FDA (**33**, Figure 18B) [168]. This overall trans-halogenation reaction was used for late-stage fluorination for the production of radiolabeled imaging reagents. Recently, different pre-targeting strategies have been developed for treatment and imaging of different diseases. Those include e.g., radiolabeling of the human A2A adenosine receptor [169], a prostate cancer-related membrane protein [170] or the combined application of biotin and tetrazine-conjugate with antibodies [171]. In all cases, it was shown that the fluorinase (FlA) accepts substrates with different moiety at C-2 of the adenine ring. So far two crystal structures of fluorinase homologs from *S. cattleya* and *Streptomyces* sp. MA37 are known and they both have a high structurally conformity [172]. In general all five known fluorinases have a high similarity of over 80% and show similar kinetic profiles [173]. Through a directed evolution approach of FlA1, different crucial amino acids for substrate binding, halide binding and hence activity were identified [174]. Additionally, the variants were tested with different unnatural substrates [175]. It was shown that the tolerance for the *wild type* (*wt*) enzyme is limited to C-2 modified substrates. However, generated variants of FlA1 also demonstrated an activity for an unnatural substrate, which were modified at C-6 positions of the adenine ring with a chlorine group [175]. These findings show that despite a narrow substrate scope of the fluorinase, it was possible to successfully apply different unnatural substrates and lay a base for directed evolution as means to use small organic compounds as substrates. However, the dependence of electrophilic substrate structures remains a drawback for the nucleophilic attack of fluoride. The crystal structure with an unnatural substrate (containing difluoromethyl groups) confirmed the necessity of geometry for activating the fluorine atom for substitution [176]. This outlines the challenge to use fluorinases as a versatile tool to generate fluorinated pharmaceutical compounds. Nevertheless, by means of designing appropriate leaving groups in combination with enzyme engineering, fluorinases could be used as tool for future generation of fluorinated pharmaceutical compounds. Data of known fluorinases are displayed in Table 5.

## 6. Conclusion on Halogens in Active Agent (Syntheses)

As we have seen in the previous paragraphs halogens are very important to many active agents as a functional moiety *per se* due to their physico-chemical properties such as bulkiness, latent polarization and as important binding partners because of halogen bonds. Organic halogen compounds are, furthermore, instrumental for synthetic purposes in terms of being good leaving groups and facilitating cross-metatheses by halogen-metal-exchanges. Nevertheless, these indispensable advantages have to be bought at a high price; namely the energy-intensive production of very toxic and hazardous chlorine gas. The reduction in energy consumption must mainly be managed by technical improvements of the chloralkali-process and enzymes are likely not able to make a significant impact. A major reason is that the majority of chlorinated compounds are necessary for different types of plastic materials (e.g., PVC) and solvents [51]. Enzymes are limitedly applicable in those areas of bulk chemicals, but there is a potential for fine chemicals. Even though halogenating enzymes will not replace conventional chlorine production, it is worth taking a look at this group of enzymes or rather at these groups of enzymes, because nature has invented these amazing enzymes at least six times. The expectations of these biocatalysts are that the conversions become environmentally more benign, the processes skip hazardous compounds such as chlorine gas and that conversions get more selective. However, the research in the field of halogenating enzymes is still at the beginning. Consistent enzymologic data such as kinetic data, measurements on stability or even well studied mutant libraries are rarely available. Many halogenating enzymes from eukaryotic sources suffer from expression challenges. Nevertheless, these enzymes open up a wide horizon of possibilities. Enormous genome data are revealing more and more halogenating enzymes and even new classes of halogenating enzymes cannot be excluded at present. Thus, there is a need for detailed and systematic research to employ halogenating enzymes for active agent synthesis, to alter their substrate scopes and enhance their process stability.

## Figures and Tables

**Table 1:** Enzymological properties of heme-dependent haloperoxidases (* original host).

Enzyme	Expression		Kinetic Parameters	Substrates
	Host	Yield [mg/L]		
*Cf*-CPO	C. fumago *	430 [67]	0.78 mm h^−1^ [59]	aromatic, alkenes
	*E. coli* BL21(DE3)	n.a. [68]		
	*Aspergillus niger*	10 [68]		
BPO	*Pseudomonas aureofaciens* [65]	n.a.	n.a. partial diastereo-selectivity [69]	aromatic, *N*-hetero-cycles, alkenes
	*Penicillus capitatus* [64,65]	n.a.		

**Table 2:** Enzymological properties of vanadium-dependent haloperoxidases.

Enzyme	Expression		Kinetic Parameters	Substrates
	Host	Yield [mg/L]		
*Ci*-V_Cl_PO	*C. inaequalis*	10 [70]	5.1 × 10^7^ M^−1^ s^−1^ for Br^−^ [75]	aromatic, alkenes
	*E. coli* BL21(DE3)	15 [76]		
	*S. cerevisiae*	100 [75]		
*Co*-V_Br_PO	*C. officinalis*		200 U/mg for MCD [83]	aromatic, *N*-hetero-cycles, alkenes
	*E. coli* BL21(DE3) (insoluble)	40		

**Table 3:** Examples of flavin-dependent tryptophan, pyrrole and phenol halogenases that can be carrier-dependent or independent.

Enzyme	Origin	Heterologous Expression Host ^1^	Product	Miscellaneous
PrnA [96]	*Pseudomonas* *fluorescens* BL915	*E. coli* ArcticExpress (DE3)	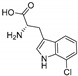	
RebH [92]	*Lechevalieria aerocolonigenes* strain 39243	*E. coli* BL21(DE3)	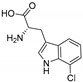	
KtzQ [110]	*Kutzneria* sp. 744		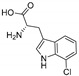	
KtzR [110]	*Kutzneria* sp. 744		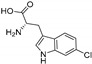	
CmdE [111]	*Chondromyces crocatus* Cm c5		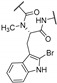	Post-NRPS (non-ribosomal peptides)
SSTH [101]	*Streptomyces toxytricini* NRRL 15443	*E. coli* BL21 CodonPlus (DE3)-RIL	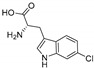	
Thal [112]	*Streptomyces* *albogriseolus*	*P. fluorescens* BL915 DORF1 and *P. chlororaphis* ACN	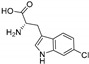	
MibH [99,113]	*Microbispora coralline* NRRL 30420		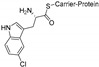	NRPS-dependent
PyrH [96,114]	*Streptomyces rugosporus* LL-42D005	*E. coli* ArcticExpress (DE3); *Pseudomonas fluorescens* BL915 ΔORF1	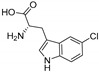	
Xcc-B B100XXXX [115]	*Xanthomonas campestris pv.* *campestris* strain B100	*E. coli* BL21(DE3) with pGro7 plasmid (Takara) for chaperone co-expression	Various substituted indoles and thereby differing regio-selectivity	
BrvH [116]	*Brevundimonas* BAL3	*E. coli* BL21(DE3) with pGro7 plasmid (Takara) for chaperone co-expression	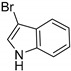	
PrnC [93,117]	*Pseudomonas* *fluorescens* BL915		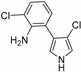	
Chl [118]	*Streptomyces aureofaciens*		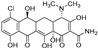	
ChlA [105]	*Dictyostelium discoideum*		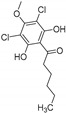	
PltA [119]	*Pseudomonas fluorescens* Pf-5	*E. coli* BL21(DE3)	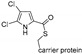	
Pyr29 [120]	*Actinosporangium vitaminophilum* ATCC 31673 and *Streptomyces* sp. Strain UC 11065		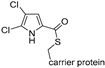	
Arm21 [121]	*Streptomyces armeniacus*		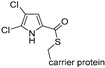	
CrpH [104]	*Nostoc* Cyanobionts		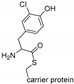	NRPS-dependent
BhaA [122,123]	*Amycolatopsis mediterranei* DSM5908		balhimycin	
SgcC3 [124]	*Streptomyces globisporus*	*E. coli* BL21(DE3) pET-30Xa/LIC	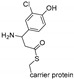	
HalB [125]	*Actinoplanes* sp. ATCC 33002	*Pseudomonas aureofaciens* ACN	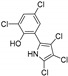	
PltM [126]	*Pseudomonas fluorescens* Pf-5	*E. coli* BL21 (DE3)	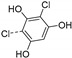	
Bmp5 [106]	*Pseudoalteromonas luteoviolacea*		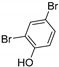	
Bmp2 [127]	*Pseudoalteromonas luteoviolacea*		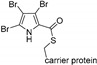	NRPS-dependent
(PltD) [13]	*Pseudomonas fluorescens* Pf-5		n.a.	Not clear if FHal
CmlS [128]	*Streptomyces venezuelae*		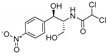	Flavin covalently bound to aspartate via CH_3_-Group
CndH [129]	*Chondromyces crocatus*		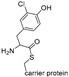	NRPS-dependent
RadH [107,130]	*Chaetomium chiversii*	*E. coli* Rosetta 2(DE3)	monocillin II	
Rdc2 [107,131]	*Pochonia chlamydosporia*	*S. cerevisiae* strain BJ5464-Npg *E. coli* BL21(DE3)	monocillin II	
TiaM [132]	*Dactylosporangium aurantiacum* NRRL 18085	*E. coli* BL21(DE3)	tiacumicin B intermediate	NRPS-dependent

^1^ if not stated otherwise, the expression took place in the origin host.

**Table 4:** Examples of heterologously expressed Fe(II)/αKG-dependent halogenases.

Enzyme	Origin/Expression Host and Yield	Features	Product/Biosynthesis
SyrB2 [139]	*Pseudomonas syringae pv. syringae* B301D/ *E. coli* strain B834(DE3) [139] n.a.	total turnover: 7 ± 2 [144]	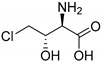 4-chloro-l-threonine/ syringomycin E
CytC3 [145]	*Streptomyces* sp./ *E. coli* BL21(DE3) [146] n.a.	n.a	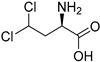 4,4-dichloro-l-valine/ dichloroaminobutyrate
WelO5 [147]	*Hapalosiphon welwitschii/* *E. coli* C43(DE3) [148,149] or BL21(DE3) [150] 20 mg L^−1^ [151]	total turnover: 75 [148] k_cat_: 1.8 ± 0.2 min^−1^ [149]	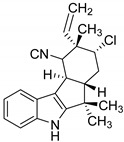 12-*epi*-fischerindole G/ fischerindole & hapalindole alkaloids
AmbO5	*Fischerella ambigua/* *E. coli* C43(DE3) [149] or BL21(DE3) [150] n.a.	k_cat_: 1.7 ± 0.1 min^−1^ [149]	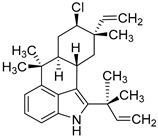 ambiguine A/ ambiguine, fischerindole and hapalindole alkaloids
WelO5* variant isoform of WelO5 (CB2) [152]	*Hapalosiphon welwitschii* IC-52-3/ *E. coli* BL21(DE3) n.a.	K_M_: 0.67 mM, k_cat_: 3.0 min^−1^	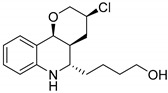 martinelline-derived fragment

**Table 5:** Examples for heterologously expressed fluorinase. Kinetic data representing the conversion of 5′-ClDA into *S*-adenosylmethionine (SAM).

Enzyme	Origin/Expression Host and Yield	Kinetic Parameters	Special Substrate Scope
FlA [165]	*Streptomyces cattleya/* *E. coli* BL21(DE3) 50 mg L^−1^ [173]	K_M_: 29.4 ± 5.80 µm k_cat_: 0.084 ± 0.005 min^−1^ [173]	[169,170,171]
FlA1	*Streptomyces* sp. MA37/ *E. coli* BL21(DE3) n.a.	K_M_: 8.36 ± 0.82 µm k_cat_: 0.13 min^−1^ [174]	
FlA4	*Streptomyces xinghaiensis/* *E. coli* BL21(DE3) n.a.	K_M_: 29.87 µm k_cat_: 0.69 ± 0.01 min^−1^ [177]	[177]

## Abbreviations

αKG	α-ketoglutarate
BPO	bromoperoxidase
CLEA	cross-linked enzyme aggregate
CPO	chloroperoxidase
FDA	5′-fluoro-5′deoxyadenosine
Fhal, FDH	flavin-dependent halogenase
FlA	fluorinase A
g	gasous
Hal	halogen
HOX	hypohalous acid
HPO	haloperoxidase
MCD	monochlorodimedone
Mn	elemental metal
MOH	metal cation hydroxid salts
NBS	*N*-bromo-succinimde
NCS	*N*-chloro-succinimide
NRPS	non-ribosomal protein synthesis
NXS	*N*-halogen-succinimide
PKS	polyketide
PVC	polyvinyl chlroide
s	solid
SAM	*S*-adenosyl methionine
X	halide ion
X_2_	molecular halogen
Y	non-halogen heteroatom
